# Supplementary material for: Universal image segmentation for optical identification of 2D materials
Source: Sci Rep. 2021 Mar 11;11:5808. doi: 10.1038/s41598-021-85159-9 (PMC7970966; doi:10.1038/s41598-021-85159-9)
Supplement: Supplementary file 1 — Supplementary Information 1. [file 41598_2021_85159_MOESM1_ESM.pdf]

Supplementary materials: Universal image segmentation for optical identification of 2D materials

Randy M. Sterbentz[1], Kristine L. Haley[1], and Joshua O. Island[1,*]

[1]Department of Physics and Astronomy, University of Nevada Las Vegas, Las Vegas, Nevada 89154, USA
[*]jisland@physics.unlv.edu

1 Python code and packages

The software was written in Python 3.7.3 and includes the following packages: scipy==1.5.2, matplotlib==3.0.3, numpy==1.16.2, and opencv-python==4.2.0.34. The full Python code is availible at https://github.com/islandlab-unlv/Universal-image-segementation.

2 Pixel accuracy and confusion matrices

In order to measure the fidelity of our program, we created confusion matrices by comparing the testing results (predictions) with manually recolored images based on layer thickness (ground truths). Figure S1(a) shows a ground truth image for a graphene flake on Si/SiO_2 and Figure S1(b) shows the testing result using the same colorscale. Figure S1(c) shows the raw counts for the confusion matrix of graphene on Si/SiO_2. Element ij in the confusion matrix is the number of pixels predicted to be i-layer that are known to be j-layer. The diagonal thus is where the program predicted the layer thickness correctly. We calculate the pixel accuracy by taking the ratio of the trace of the matrix with the sum of all elements in the matrix. For graphene on SiO_2, we calculate a pixel accuracy of 96.7%. For the other test samples in Figure S1, we calculate the following pixel accuracies: MoS_2/SiO_2 in Figure S1(e-h) (94.7%), MoS_2/PDMS in Figure S1(i-l) (98.0%), $MoSe_2$/PDMS in Figure S1(m-p) (96.7%).

Normalizing a confusion matrix by the true labels elucidates how well the program can predict each layer thickness. Columns 0-2 of Figure S1(d), for example, state we can successfully identify 99.0% of the Si/SiO_2 substrate, 97.3% of the monolayer graphene regions, and 94.3% of the bilayer graphene regions. It appears to falter with tri- and quadlayer identification, but this is a result of the necessary preprocessing on the test image. Despite qualities of bilateral filtering as an edge-preserving filter, we always had some blurring at the flake-thickness boundaries while applying it. As a result, narrow strips of a layer thickness could be blurred away, as is the case with the thin stripes of trilayer present in the graphene/SiO_2 test image (light grey stripes at the bottom left of panels (a) and (b) of Figure S1). These confusion matrices were created off of a single test image per material/substrate tested, and are beholden to the biases that arise from those individual samples. With finer tuning of the bilateral filter and a larger test set, this blurring should be mitigated.

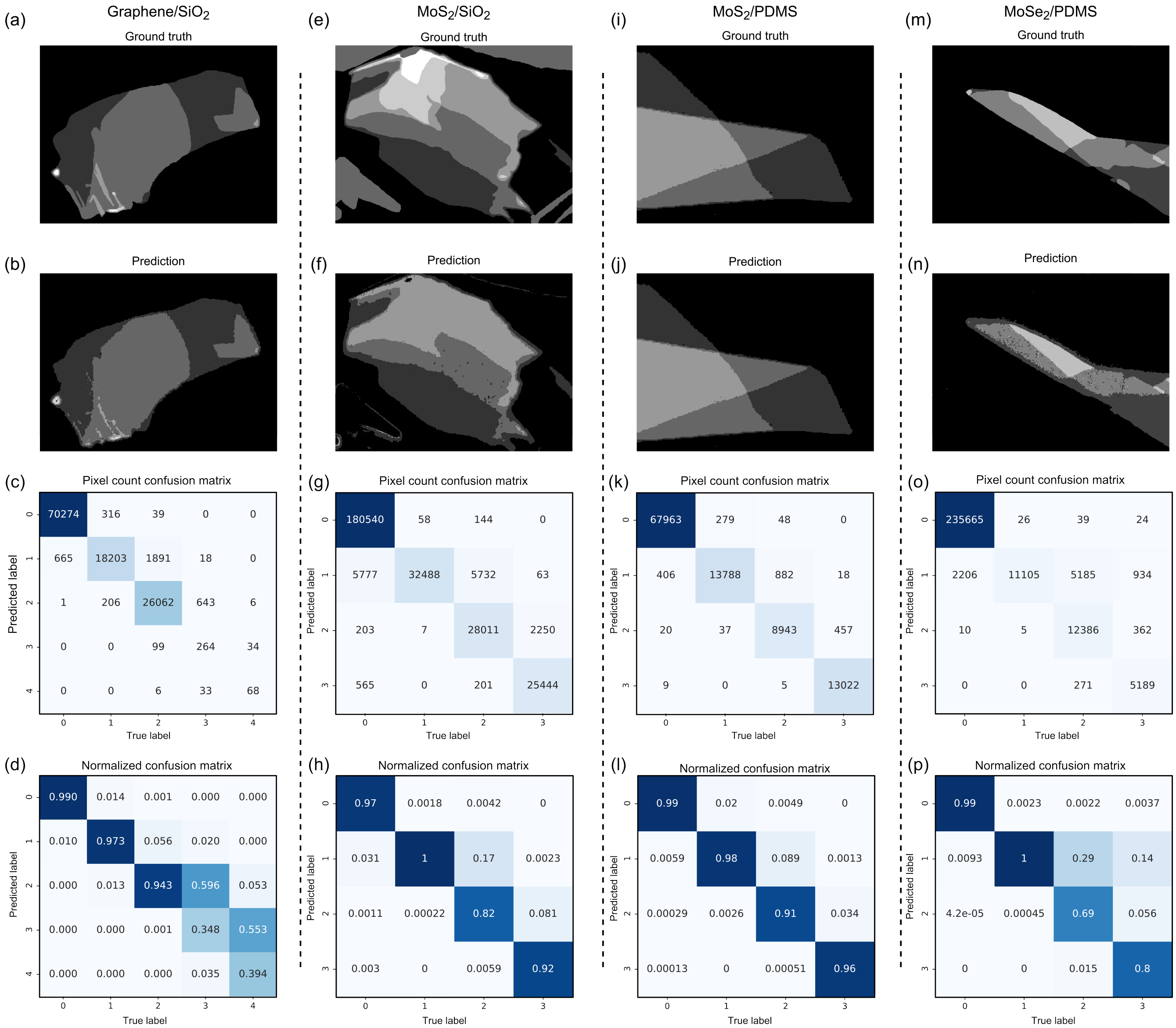

Figure S1. Pixel accuracy testing. (a-d) Ground truth (a) and prediction (b) images from testing for a graphene flake on a Si/SiO_2 substrate. Raw pixel count confusion matrix for graphene on SiO_2 (c), and the same confusion matrix normalized by column (normalized to true label) (d). (e-h) Ground truth (e) and prediction (f) images from testing for a MoS_2 flake on a Si/SiO_2 substrate. Raw pixel count confusion matrix for MoS_2 on SiO_2 (g), and the same confusion matrix normalized by column (normalized to true label) (h). (i-l) Ground truth (i) and prediction (j) images from testing for a MoS_2 flake on a PDMS substrate. Raw pixel count confusion matrix for MoS_2 on PDMS (k), and the same confusion matrix normalized by column (normalized to true label) (l). (m-p) Ground truth (m) and prediction (n) images from testing for a $MoSe_2$ flake on a PDMS substrate. Raw pixel count confusion matrix for $MoSe_2$ on PDMS (o), and the same confusion matrix normalized by column (normalized to true label) (p).
